# CTNNB1 syndrome mouse models

**DOI:** 10.1007/s00335-025-10105-3

**Published:** 2025-01-20

**Authors:** Duško Lainšček, Vida Forstnerič, Špela Miroševič

**Affiliations:** 1 Department of Synthetic Biology and Immunology, National Institute of Chemistry, Ljubljana, 1000 Slovenia; 2 Centre for Technologies of Gene and Cell Therapy, Ljubljana, 1000 Slovenia; 3 EN-FIST Centre of Excellence, Ljubljana, 1000 Slovenia; 4 The Gene Therapy Research Institute, CTNNB1 Foundation, Ljubljana, 1000 Slovenia; 5 Department of Family Medicine, Faculty of Medicine Ljubljana, University of Ljubljana, Ljubljana, 1000 Slovenia

**Keywords:** CTNNB1 syndrome, Β-catenin, Rare disease, Mouse model, Neurodevelopmental disorder, Gene therapy, Cre-loxp

## Abstract

CTNNB1 syndrome is a rare neurodevelopmental disorder, affecting children worldwide with a prevalence of 2.6–3.2 per 100,000 births and often misdiagnosed as cerebral palsy. *De novo* loss-of-function mutations in the *Ctnnb1* gene result in dysfunction of the β-catenin protein, disrupting the canonical Wnt signaling pathway, which plays a key role in cell proliferation, differentiation, and tissue homeostasis. Additionally, these mutations impair the formation of cell junctions, adversely affecting tissue architecture. Motor and speech deficits, cognitive impairment, cardiovascular and visual problems are just some of the key symptoms that occur in CTNNB1 syndrome patients. There is currently no effective treatment option available for patients with CTNNB1 syndrome, with support largely focused on the management of symptoms and physiotherapy, yet recently some therapeutic approaches are being developed. Animal testing is still crucial in the process of new drug development, and mouse models are particularly important. These models provide researchers with new understanding of the disease mechanisms and are invaluable for testing the efficacy and safety of potential treatments. The development of various mouse models with β-catenin loss- and gain-of-function mutations successfully replicates key features of intellectual disability, autism-like behaviors, motor deficits, and more. These models provide a valuable platform for studying disease mechanisms and offer a powerful tool for testing the therapeutic potential and effectiveness of new drug candidates, paving the way for future clinical trials.

## Introduction

CTNNB1 syndrome, a rare autosomal dominant neurodevelopmental disorder, is caused by pathogenic loss-of-function (LoF) variants in the *Ctnnb1* gene, which encodes the β-catenin protein, a critical regulator of the Wnt/β-catenin signaling pathway (Liu et al. 2022; Zhuang et al. 2023). LoF mutations in the *Ctnnb1* gene disrupt processes in this pathway, leading to a wide range of symptoms across multiple domains. Neurological and motor impairments are prominent, with patients exhibiting muscle weakness, hypotonia, spasticity, and developmental delays; many achieve walking milestones late or rely on wheelchairs. Intellectual and developmental disabilities are common, ranging from mild to severe, and are often accompanied by speech, language, and behavioral challenges, including autism spectrum disorder (ASD) and anxiety. Visual impairments, such as strabismus, are frequent. Dysmorphic facial features, including a broad nasal tip and thin upper lip, are noted in the majority of cases, potentially aiding earlier diagnosis. Genetic testing remains essential, with phenotypic recognition and improved diagnostic tools offering hope for timely identification and intervention. MRI often reveals nonspecific white matter abnormalities, while EEG findings are typically unremarkable (Miroševič et al. 2022; Kayumi et al. 2022; Sinibaldi et al. 2023; Sudnawa et al. 2024; Garone et al. 2024). The first report linking *Ctnnb1* mutations to intellectual disability (ID) was published in 2012 (de Ligt et al. 2012), followed by the identification of *de novo* mutations associated with a recognizable syndrome featuring autistic traits in 2014 (Dubruc et al. 2014; Kuechler et al. 2015), whereas the syndrome was first identified in a study by Tucci et al. who showed an impact of β-catenin mutations on neurodevelopmental features in a mouse model which correlated with patient phenotypes (Tucci et al. 2014).

CTNNB1 syndrome is frequently misdiagnosed as cerebral palsy due to overlapping motor symptoms, leading to underdiagnosis worldwide (Jin et al. 2020; Moreno-De-Luca et al. 2021). The disorder affects both genders equally, with a prevalence of 2.6–3.2 per 100,000 live births (López-Rivera et al. 2020). Currently, there is no cure, and available therapies are limited to symptom management and physiotherapy but just recently some of the researchers started to develop potential cure for the CTNNB1 syndrome, relying on small chemical inhibitors or on the AAV based gene replacement therapy. *Ctnnb1* is now recognized as a high-risk gene for autism spectrum disorder (ASD), and it is categorized as a Category 1 ASD-risk gene in the SPARK Gene List and the SFARI Gene Database (Zhuang et al. 2023).

Human *Ctnnb1* gene (ENSG00000168036, OMIM 116806) is located at chromosome 3p22.1 (GRCh38; chr3: 4199505–41240443; from omim.org) and has 63 transcripts, 218 orthologous, 4 paralogues and is connected to 150 different phenotypes (ensemble.org). Mouse *Ctnnb1* gene (ENSMUSG00000006932) is located at chromosome 9 (GRCm39; chr9: 120758282–120789573; from ensemble.org) and has 15 transcripts, 218 orthologues, 4 paralogues and is associated with 132 phenotypes. The *Ctnnb1* gene in both mice and humans typically consists of 14 protein-coding exons that produce the β-catenin protein, which is composed of 781 amino acids.

The β-catenin protein belongs to the armadillo family of structural proteins and is involved in both embryonic development and adult homeostasis. Multiple studies highlight the significance of β-catenin in successful embryo implantation and in organ development (Huelsken et al. 2000; Rudloff and Kemler 2012; Messerschmidt et al. 2016; Chalamalasetty et al. 2016; Ostrin et al. 2018). The β-catenin protein has a dual function in both Wnt signaling and cell-cell adhesion. Structural and signaling roles of β-catenin are mutually exclusive, which is reflected in its protein structure. β-catenin consists of three regions: an unstructured N-terminal region important for degradation, a C-terminal region involved in transcriptional activity and stability, and a highly conserved central core with 12 armadillo repeats (ARM) that interact with over 20 protein partners, including E-cadherin and TCF (Huber et al. 1997; Mo et al. 2009; Tian et al. 2011). Interactions with intracellular cadherin domains occur throughout the whole central armadillo-repeat region of β-catenin, integrating processes such as cell adhesion, cell migration, neurite outgrowth, and synaptic remodeling (Tucci et al. 2014).

**Fig. 1:**
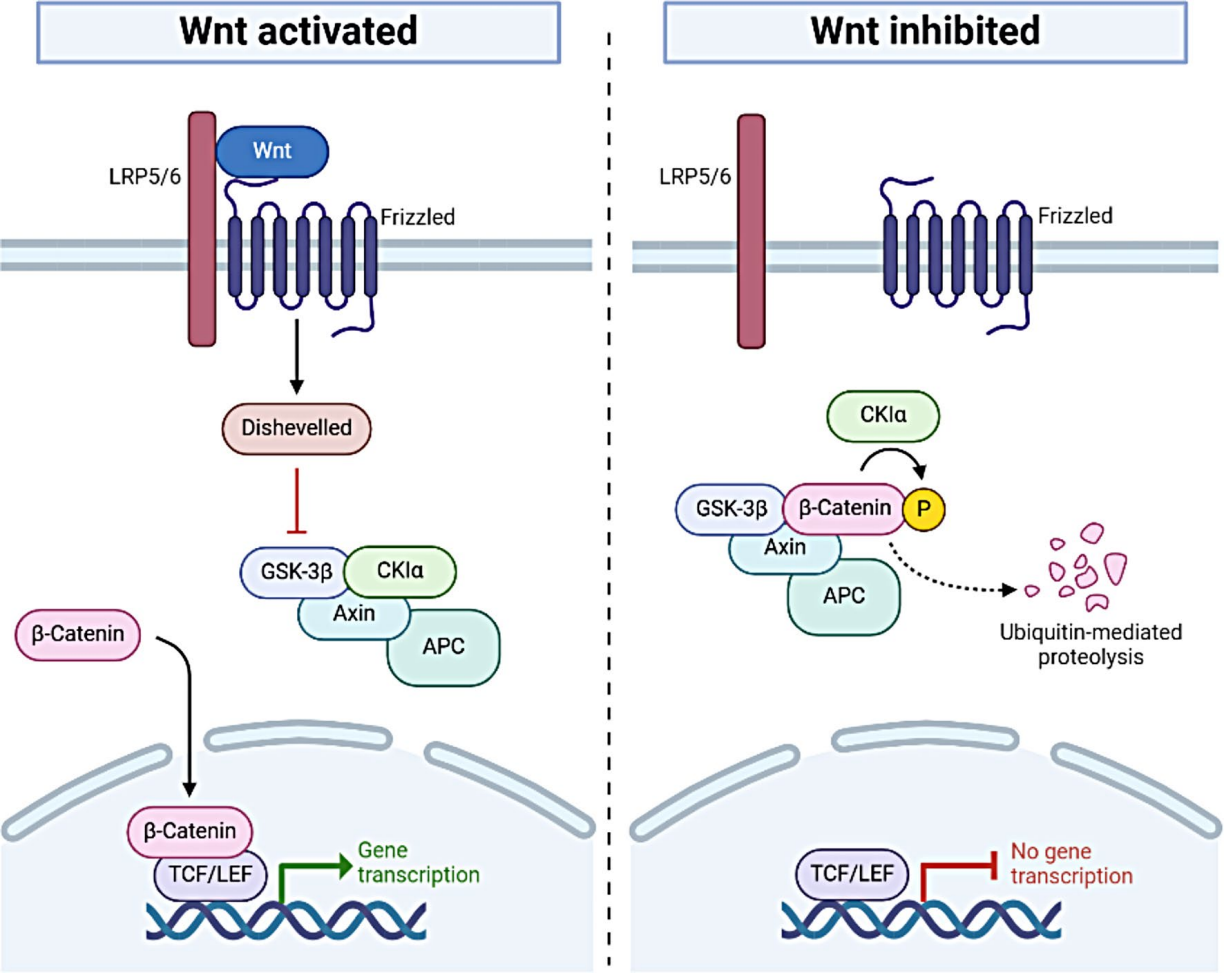
The canonical Wnt/β-catenin signaling pathway. When the Wnt pathway is activated, the Wnt ligand binds to the Frizzled receptor and LRP5/6, recruiting Dishevelled (DVL), which disrupts the formation of the destruction complex (GSK-3β, CK1α, Axin, and APC). This prevents β-catenin degradation, allowing it to accumulate in the cytoplasm and translocate to the nucleus, where it acts as a co-activator of LEF1/TCFs to promote gene transcription. In the absence of Wnt signaling, the destruction complex phosphorylates β-catenin, leading to its ubiquitination by the SCF complex and subsequent degradation via the proteasome

Cytosolic β-catenin levels are tightly regulated by degradation through the destruction complex (Fig. 1). In the absence of Wnt signaling, newly synthesized β-catenin is continuously degraded, with the remaining non-degraded β-catenin binding to E-cadherin and α-catenin at the cell membrane to form adhesion complexes. The destruction complex, comprising axin 1, APC, CK1, and GSK3β, phosphorylates β-catenin, marking it for ubiquitination and proteasomal degradation. When the canonical Wnt pathway is activated, Wnt ligands bind Frizzled receptors, prompting Dishevelled to sequester the destruction complex and prevent β-catenin degradation (Kimelman and Xu 2006; Schwarz-Romond et al. 2007). This allows β-catenin to accumulate, translocate to the nucleus, and activate the Wnt transcriptional program by interacting with TCF-LEF transcription factors, regulating genes such as MYC and CCND1 involved in cell proliferation, differentiation, and inflammation (Boyer et al. 2021; Ryner et al. 2023; Chen et al. 2023; Cai et al. 2024).

While gain-of-function (GoF) mutations in *Ctnnb1* are linked to cancers such as liver and colorectal cancer (Harada et al. 1999; Veelen et al. 2011; Singh et al. 2014; Zhang et al. 2021; Loesch et al. 2022; Cai et al. 2024), autosomal *de novo* loss-of-function mutations can lead to CTNNB1 syndrome, a neurodevelopmental disorder (NDD; Phenotype MIM Number 615075) (Kuechler et al. 2015; Kayumi et al. 2022; Basu et al. 2024).

Currently, CTNNB1 syndrome has no cure, and treatment options are primarily focused on alleviating symptoms. Therefore, finding new drugs to help those affected by this rare genetic disorder is essential. In the study of new diseases and the development of therapies, animal models are critically important (Harris 2021). Given the high similarity between the human and mouse genomes (approximately 97.5%) (Breschi et al. 2017), mouse models are frequently utilized. Since CTNNB1 syndrome arises from loss-of-function mutations that result in the loss of functional β-catenin protein, creating mouse models that replicate the syndrome’s symptoms is vital. Based on the Mouse Genome Informatics (MGI) database (ID MGI:88276) 36 alleles were generated that result in the modification of the *Ctnnb1* gene in mouse models, however, some models lack clear phenotype annotation, whereas some are not yet included in the MGI database. In the present study we review mouse models with alterations in the *Ctnnb1* gene, including gain- (GoF) and loss-of-function (LoF) models, focusing on mouse models exhibiting symptoms of neurodevelopmental disorders, including models with aberrant brain/embryo development, and motor disorders, both hallmarks of the CTNNB1 syndrome. In the category of GoF mouse models, the foundational mouse model most often employed for generation of subsequent GoF models was the *Ctnnb1*^*tm1Mmt*^ mouse (Harada et al. 1999) in which exon 3, which contains phosphorylation sites that regulate the degradation of β-catenin, was sandwiched by loxP sites, highlighting the role of Wnt signaling in intestinal tumorigenesis. A pioneering LoF model is the *Ctnnb1*^*tm2Kem*^ model (Brault et al. 2001), in which exons 2–6, corresponding to the protein region between the unstructured N-terminal part to the middle of ARM3, were targeted resulting in an embryonic lethal phenotype. In relation to CTNNB1 syndrome, it is noteworthy that the chemically induced mouse model *Bfc+* (Tucci et al. 2014) was the first to link features of the CTNNB1 syndrome phenotype to mutations in the *Ctnnb1* gene.

## Loss-of function *Ctnnb1* mouse models

Using Cre DNA recombinase in conjunction with its loxP recognition sites allows for conditional gene function studies in adult mice or specific cell types and tissues when Cre is expressed under tissue- or cell-specific promoters. By inserting a targeted gene between two loxP sites, Cre-mediated excision of the gene of interest can occur. Depending on the promoter-driven expression of Cre recombinase, either whole-body gene inactivation or cell- or tissue-specific conditional knockout mouse models can be generated (Schnütgen et al. 2003; Friedel et al. 2011). Both conditional and conventional knockout mouse models relevant to *Ctnnb1* loss-of-function are summarized in Table 1.

**Table 1:** Overview of key *Ctnnb1* LoF mouse models relevant to CTNNB1 syndrome studies

Allele	Synonyms	Type	Tissue/Cell type	Reference
*Ctnnb1* ^*tm1Max*^	beta-cat -, beta-catenin^n^, Catnb^tm1Kem^	KO	Whole body	(Haegel et al. 1995)
*Ctnnb1* ^*tm1Wbm*^	beta-cat^del^	KO	Whole body	(Huelsken et al. 2000)
*Ctnnb1* ^*tm2Wbm*^	beta-cat^lacZ^(neo)	KO	Whole body	(Huelsken et al. 2000)
*Ctnnb1* ^*tm3Wbm*^	beta-cat^lacZ^(hygro)	KO	Whole body	(Huelsken et al. 2000)
*Ctnnb1* ^*tm4Wbm*^	beta-catenin^flox^, beta-catenin^lox^, beta-cat lox, beta-cat^loxEx3–6^, Ctnnb1^fl.^	KO	Whole body	(Huelsken et al. 2000)
*Ctnnb*^*1tm2.1Kem*^ +Wnt1-Cre	beta-catenin^del^, Ctnnb1^Del^, floxdel	cKO	Developing neural tube	(Brault et al. 2001)
*Ctnnb1*^*tm2Kem*^ + Wnt1-Cre	B-catenin^fl2–6^, BcatLOF, Beta-Cat^c^, beta-catenin^c^, beta-catenin^deltaex2–6−fl^, beta-catenin^f^, beta-catenin^fl.^, beta-catenin^flox^, beta-catenin^floxed^, beta-catenin^lox^, beta-catenin/loxP(ex2-6), beta-cat^ex2–6^, beta-cat^fl.^, beta-Cat^flox^, beta-cat^lof^, betaCatN, beta-Ctn^fl.^, Catnb1^tm2Kem^, Catnb^fx^, Catnb^lox(ex2–6)^, Catnb^tm2Kem^, Catnb^tm2Kwem^, Ctnb^floxE2−E6^, Ctnnb1^f^, Ctnnb1^fl.^, Ctnnb1^flox^, Ctnnb1^floxed^, Ctnnb1^fx^, Ctnnb1^loxp^	cKO	Developing neural tube	(Brault et al. 2001)
*Ctnnb1*^*tm4Wbm*^ + Brn4-Cre		cKO	CNS	(Zechner et al. 2003)
*Ctnnb1*^*tm1Max*^ *+* D6-Cre		cKO	CNS (cerebral cortex, hippocampus)	(Machon et al. 2003)
*Ctnnb1*^*tm1.1Yy*^+Col2a1-Cre	Catnb^−^	cKO	Motor system	(Guo et al. 2004)
*Ctnnb1*^*tm1Max*^ *+* Emx1-Cre		cKO	CNS (cerebral cortex, hippocampus)	(Campos et al. 2004)
*Ctnnb1*^*tm2Kem*^ + Nes8-Cre		cKO	CNS (telencephalon)	(Mattias Backman et al. 2005)
*Ctnnb1*^*tm2Kem*^ *+* Nestin-Cre	Nestin-cre,β-catenin^Fl/+^	cKO	CNS (cerebellum)	(Schüller and Rowitch 2007)
*Ctnnb1*^*tm2Kem*^ *+* Hsa-Cre		cKO	Skeletal muscles	(Li et al. 2008)
*Ctnnb1*^*tm2Kem*^ *+* HB9-Cre		cKO	Motoneurons	(Li et al. 2008)
*Ctnnb1*^*tm1Kba*^ + Wnt1-Cre	Beta-catenin^D164A^	cKO	Embryo development	(Valenta et al. 2011)
*Ctnnb1*^*tm2Kba*^ + CMV-Cre	Beta-catenin^deltaC^	cKO	Embryo development	(Valenta et al. 2011)
*Ctnnb1*^*tm3Kba*^ + Wnt1-Cre	Beta-catenin^D164A−deltaC,^ Beta-catenin^dm^, Ctnnb1^dm^	cKO	Embryo development	(Valenta et al. 2011)
*Ctnnb1*^*tm2Kem*^ + Pvalb-Cre		cKO	Parvalbumin interneurons	(Dong et al. 2016)
*Ctnnb1*^*tm1Knw*^ + Zp3-Cre	betaCatC^f^	cKO	Embryo implantation	(Messerschmidt et al. 2016)
*Ctnnb1*^*tm2Kem*^ *+* Foxd1-Cre		cKO	Hypothalamic and prethalamic neuroepithelium	(Newman et al. 2018a)
*Ctnnb1*^*tm2Kem*^ *+* Nkx2.1-Cre		cKO	Hypothalamic and prethalamic neuroepithelium	(Newman et al. 2018a)
*Ctnnb1*^*tm2Kem*^*/ +* CamKIIα-Cre		cKO	Forebrain neurons	(Wickham et al. 2019)
*Ctnnb1*^*tm2Kem*^ *+* E2A-Cre		KO	Whole body	(Alexander et al. 2024)

KO-knockout; cKO-conditional knockout; CNS-central nervous system

Defects in several mouse genes encoding components of the cadherin-catenin system have been shown to cause defects in embryogenesis. Mutations in the *Ctnnb1* gene in humans are limited to heterozygous or mosaic mutations and it was shown quite early on that homozygous loss-of-function mutations in mice also have an embryonic lethal effect. To study the effect of β-catenin as the central protein of the highly conserved Wnt pathway, two groups generated null mice by classic transgenesis via homologous recombination in ES cells wherein DNA segments containing exon 2 and partial exon 3 (Haegel et al. 1995) or most of exon 3 and exon 4 (Huelsken et al. 2000) were replaced with a neomycin resistance cassette resulting in no detectable RNA or protein levels of β-catenin. In both cases, heterozygous mutant animals derived from the ES clones were bred on a mixed 129xC57Bl6 background, and showed no developmental abnormalities. By observing mutant mouse embryos, a Mendelian ratio of β-catenin–deficient embryos was observed up to E7.5 of development, but the ratio was reduced to 9% at E8.5 and after E9.5 no homozygous mutant embryos were present, demonstrating that homozygote mutations are lethal due to effects on ectoderm and mesoderm formation and defects in anterior-posterior axis formations. Notably, β-catenin-deficient embryos retained functional adherens junctions, as plakoglobin (γ-catenin), another member of the armadillo protein family, was able to compensate for β-catenin’s role in cell adhesion (Haegel et al. 1995; Huelsken et al. 2000).

The first conditional *Ctnnb1* heterozygous knockout was made by Brault et al., in which *Ctnnb1* KO was restricted to regions of *Wnt1* expression by encoding Cre recombinase under the control of *Wnt1* regulatory sequences (Brault et al. 2001). Two loxP sites were introduced between exon 2 and 6 of the β-catenin gene to generate floxed mice. First, *Wnt1-Cre* transgenic mice were crossed with mice heterozygous for the β-catenin floxdel allele (C57/BL6 background). The offspring that inherited both the *Wnt1-Cre* transgene and a floxdel allele were then bred with homozygous floxed β-catenin mice. The resulting strain carrying the *Wnt1-Cre* transgene along with one floxed and one floxdel allele enabled targeted deletion of *Ctnnb1*. Initial findings concerning homozygous lethality due to the anterior-posterior axis formations malformations were confirmed by Brault et al. who also found that homozygous embryos died at gastrulation. Extensive deformations in brain morphogenesis and craniofacial structures were present. Histological analysis revealed that E12.5 embryos had no discernible midbrain, neither a cerebellum nor a choroid plexus, forebrain was not developed properly, craniofacial structures were absent and most of the cranial bones were missing, confirming the importance of β-catenin/Wnt in neuronal development. Additionally, higher levels of apoptosis were identified in neural crest cells via TUNEL assay (Brault et al. 2001).

The importance of β-catenin/Wnt signaling in proliferation of neuronal progenitors, determining the size of the progenitor cell pool, and dictating neuronal progenitors to proliferate or to differentiate was confirmed also in embryos of mice with ablated *Ctnnb1* expression in the developing CNS (central nervous system) (Zechner et al. 2003). Conditional KO targeting floxed *Ctnnb1* exons 3–6 with Cre recombinase under the control of the neural specific Brn4 promoter showed decreased size of the spinal cord compared to wild-type embryos, additionally ventricles were missing at E12. Lower proliferation and increased apoptosis (by 300% at E11.5 and E13) of neuronal progenitors was again confirmed, leading to overall smaller size and underdevelopment of the nervous system (Zechner et al. 2003).

Additional models confirming the importance of *Ctnnb1* in embryo development were developed by Valenta et al. and Messerschmidt et al. (Valenta et al. 2011; Messerschmidt et al. 2016) all on a C57/BL6 background. Valenta et al. generated three distinct mouse models derived from the *Ctnnb1*^*tm2Kem*^ mouse to investigate the role of different regions of β-catenin in embryonic development. Their research demonstrated that the C-terminal region is essential for mesoderm formation and normal gastrulation, while the N-terminal region is crucial for later stages of embryonic development. The first model, *Ctnnb1*^*D164A*^, introduced a point mutation in exon 4, resulting in a D164A substitution within the first Armadillo repeat. This mutation disrupted binding to the N-terminal coactivator BCL9/BCL9L, and homozygous embryos showed developmental arrest around E10.0, consistent with observations by Haegel et al. and Huelsken et al. The second model, *Ctnnb1*^*deltaC*^, introduced a premature stop codon at position 673 in exon 13, producing a C-terminal truncated β-catenin protein. To eliminate the possibility of RNA-mediated decay, exon 15 was fused directly to exon 13. Homozygous embryos failed to progress beyond gastrulation and died at E7.5. The third model, *Ctnnb1*^D164A − deltaC^, combined the D164A point mutation with the C-terminal truncation. Homozygosity in this double mutant also resulted in embryonic lethality during gastrulation at E7.5. These findings underscore the indispensable roles of both the N-terminal and C-terminal regions of β-catenin in embryogenesis, with homozygous mutations proving lethal at distinct developmental stages depending on the affected region.

A significant study demonstrating the crucial role of β-catenin in embryo development and implantation was conducted by Messerschmidt et al. (Messerschmidt et al. 2016). They developed the *Ctnnb1*^*tm1Knw*^ conditional mouse model, which deletes exons 8–13, truncating the C-terminal region of the β-catenin protein. Homozygous mutants were not viable, while heterozygous mice were viable and fertile. To investigate the impact of this novel allele on embryo implantation, they used Zp3-Cre to conditionally delete *Ctnnb1* in growing oocytes (de Vries et al. 2000). Their findings revealed that phenotypic differences appeared earlier than previously predicted by Haegel et al. and Huelsken et al., not during gastrulation but at the blastocyst stage. The truncation of β-catenin disrupted normal cell adhesion within the blastocyst, leading to embryo fragmentation. This study conclusively showed that loss of β-catenin function impairs embryo implantation by preventing proper blastocyst development (Messerschmidt et al. 2016).

Together, it can be concluded that full body homozygous KO of C*tnnb1* results in an embryonic lethal phenotype as does conditional KO in CNS and that β-catenin has a crucial role in proliferation and the balance between progenitor expansion and differentiation in the developing nervous system therefore using mouse models with full body C*tnnb1* KO is useful to study the impact of β-catenin on embryo and body development. While these mouse models provide critical insight into the developmental role of *Ctnnb1*, they are not useful in the context of therapy development for CTNNB1 syndrome, since no viable offspring are produced, therefore using heterozygous animals that exhibit some phenotypic differences from their wild-type littermates are needed. These strains have however been further utilized (especially Brault 2001) for obtaining several conditional KO models which further establish the role of β-catenin in specific cells and tissues and to some extent mirror some characteristics seen in CTNNB1 syndrome patients.

To determine the role of canonical Wnt signaling in the development of the hypothalamus and prethalamus Newman et al. developed two conditional *Ctnnb1* knockout animals (Newman et al. 2018b). The background for the generation of cKOs were *Ctnnb1* floxed mice (Brault et al. 2001). To specifically delete the *Ctnnb1* gene in posteroventral hypothalamus a specific strain of posteroventral hypothalamic Nkx2.1-Cre transgene mice were used (Shimogori et al. 2010), whereas for targeting the *Ctnnb1* deletion within the hypothalamic and prethalamic neuroepithelium Foxd1-Cre mice were employed (Newman et al. 2018a). Deletion of the *Ctnnb1* gene resulted in an anteriorized and hypoplastic hypothalamus whereas posterior structures were lost or reduced, further confirming the importance of Wnt signaling in brain development.

Schüller et al. also confirmed through use of Nestin-Cre mice (Tronche et al. 1999) leading to a cerebellum tissue conditional ablation of the *Ctnnb1* gene, that β-catenin is crucial for normal brain development as it influences normal morphogenesis of the caudal midbrain and the cerebellum (Schüller and Rowitch 2007). Similar detrimental effects on cerebral cortex and hippocampus formation due to the lack of β-catenin were presented by Campos et al., where ablation was carried out by the Emx1-Cre mice line (Campos et al. 2004). These findings were also confirmed by Machon et al. by using D6-Cre cKO, where *Ctnnb1* conditional inactivation in the mouse cerebral cortex and hippocampus occurs after embryonic day (E) 10.5 (Machon et al. 2003). Ablation of β-catenin under Nes8-Cre expressing manner also effect telencephalon development (Mattias Backman et al. 2005).

A similar finding regarding the importance of β-catenin in brain development was confirmed by Wickham et al., who generated conditional knockout animals with the *Ctnnb1* gene inactivated specifically in forebrain neurons. The mice were generated through mating *Ctnnb1* floxed mice (Brault et al. 2001) and CamKIIα-Cre mice (Rios et al. 2001). The Cre recombinase is expressed during synaptogenesis with full activation by postnatal day 21, in cortical and hippocampal excitatory glutamatergic neurons (Rios et al. 2001; Mohn et al. 2014; Pirone et al. 2017), resulting in abundant reduction of β-catenin levels in the hippocampus and cortex. Over a 7-day trial, these mice demonstrated cognitive impairments, failing to reach the same freezing levels as controls and struggling to learn the goal location in the Barnes Maze. Motor abilities remained intact, as evidenced by normal performance in the rotarod test and consistent exploration distances. Behavioral tests showed no signs of autism spectrum disorder (ASD), as the mice performed normally in marble burying and social interaction tests. However, anxiety-like behavior was evident: the mice were less inclined to explore new surroundings in the open field test and spent less time in the lit box during the dark/lit box assay. Molecular analysis revealed reduced levels of N-cadherin, α-N-catenin, p120ctn, and S-SCAM/Magi2 in cKO animals, while canonical Wnt target genes were unaffected. As observed in earlier studies (Haegel et al. 1995; Huelsken et al. 2000), an upregulation of γ-catenin (plakoglobin) was detected, likely partially compensating for the role of β-catenin in cell adhesion. Overall, while mice showed significant cognitive impairments, mirroring the intellectual disability characteristic of CTNNB1 syndrome, they lacked motor impairments or autism-like behaviors, making the model unsuitable for studying motor deficits or ASD, therefore this mouse model is not a perfect model for determining the therapeutic effect of potential new therapy.

CTNNB1 syndrome patients often exhibit motor impairments, including severe spasticity. This was explored by Li et al., who demonstrated that disrupting *Ctnnb1* using a skeletal muscle-specific Cre line (Hsa-Cre) led to neuromuscular junction dysfunction. Notably, acetylcholine receptor clusters were enlarged and distributed over a broader region, while primary nerve branches were mislocated, and secondary intramuscular nerve branches were elongated but fewer in number. Additionally, both spontaneous and evoked neurotransmitter release were reduced, with compromised short-term plasticity and calcium sensitivity of release in β-catenin-deficient muscle. Interestingly, these effects were absent when *Ctnnb1* was deleted via HB9-Cre, a motor neuron-specific promoter, emphasizing the role of muscle-specific β-catenin loss in these abnormalities (Li et al. 2008).

Alexander et al. generated a mouse model for *Ctnnb1* haploinsufficiency (Alexander et al. 2024) by crossing β-cat^fl/fl^ (loxP exons 2–6 of the *Ctnnb1* gene) (Brault et al. 2001) and Ella/E2a-Cre (E2A-Cre) mice that expresses Cre recombinase under the adenoviral EIIa promotor, which targets the Cre recombinase to the early mouse embryo (Lakso et al. 1996). The resulting mice strain is a hybrid mix of C57B6/J, 129/ SvJ and FVB mice strains. The β-catenin heterozygous mice displayed a 50% reduction in β-catenin protein levels and exhibited both motor and cognitive impairments characteristic of CTNNB1 syndrome and can be very useful in CTNNB1 syndrome therapy validation. Reduced synaptic adhesion was evident from diminished interactions between N-cadherin and β-catenin, though LEF1 levels remained unchanged. Behavioral assessments revealed cognitive deficits: during contextual fear conditioning and probe trial assays, the mice showed reduced freezing behavior both during training and in the 24-hour probe trial for short-term memory. Motor impairments were evident from reduced forelimb grip strength and poor performance on the rotarod test. However, locomotor activity appeared unaffected, as the open field assay showed no significant differences in distance traveled between genotypes. Additionally, the study identified changes in hippocampal neuron excitability and altered levels of Na/K ATPases (Alexander et al. 2024).

One of the characteristics of CTNNB1 syndrome is the presence of autism spectrum disorder (ASD). *De novo* mutations are notably concentrated in genes associated with the Wnt pathway, indicating that Wnt signaling may serve as a convergence point for genetic risk factors in ASD (O’Roak et al. 2012). Supporting this hypothesis, several mouse models with deletions in Wnt pathway genes exhibit impairments in social interaction and display repetitive behaviors. To understand how *Ctnnb1* deficiency contributes to ASD, Dong et al. created a *Ctnnb1* conditional knockout specifically in parvalbumin (PV) interneurons, which represent a major neuron type that mediates ASD pathophysiology (Dong et al. 2016). *Ctnnb1*^flox/flox^ mice (B6.129-*Ctnnb1*^*tm2Kem*^/*KnwJ*) with loxP sites flanking exons 2–6 of *Ctnnb1* (Brault et al. 2001) and PV-Cre mice (B6; 129P2-Pvalb^*tm1(cre)Arbr*^/J) (Hippenmeyer et al. 2005) from the Jackson Laboratory (Bar Harbor, ME) were used to generate this heterozygous strain. The PV-Cre;*Ctnnb1*^flox/flox^ mouse model was generated by F1 hybrid backcrossing. The mutant mice exhibited a complex behavioral profile, combining anxiety-like behaviors, ASD traits, and selective cognitive impairments, making it suitable for studying these features of CTNNB1 syndrome. Despite no motor deficits observed in open field and rotarod tests, mutant mice displayed significant anxiety, evident in reduced center exploration in open field tests, shorter open-arm exploration in the elevated plus maze, prolonged grooming, and increased head-dipping.

Cognitively, the mice showed mixed results: impaired object recognition and social interactions, long-term memory deficits, and repetitive behaviors characteristic of ASD, but enhanced spatial memory in the Morris water maze. Altered neuronal activity and a higher percentage of PV + neurons in the prefrontal cortex were observed, while other brain regions showed no PV + changes. While ASD-like symptoms are evident in some GoF models, these findings suggest that ASD symptoms may result also from loss-of-function mutations in *Ctnnb1*. Together, this model provides valuable insights into ASD, anxiety, and cognitive impairments associated with CTNNB1 syndrome (Dong et al. 2016), thus can be suitable for therapeutic efficacy determination.

## Gain-of function *Ctnnb1* mouse models

Mouse models with enhanced β-catenin/Wnt signaling are described as gain-of function mouse models and are presented in Table 2.

**Table 2:** Overview of key *Ctnnb1* GoF mouse models relevant to CTNNB1 syndrome studies

Allele	Synonyms	Type	Tissue/Cell type	Reference
*Ctnnb1* ^*tm1Mmt*^	B-catenin^fl3^, BcatGOF, beta-cat^act^, beta-cat^c.a^., beta-catenin^deltaex3^, beta-catenin^lox−ex3^, beta-catenin^loxEx3^, beta-catenin/loxP(ex3), beta-cat^ex3^, beta-cat^gof^, beta-cat^loxEx3^, Catnb^(ex3)fl^, Catnb^(Flox3^, Catnb^lox(ex3)^, Catnb^tm1Tak^, Ctnnb1^ex3^, Ctnnb1^(Ex3)fl^, Ctnnb1^ex3(loxP)^, Ctnnb1^f(Ex3)^, Ctnnb1^fl(ex3)^, Ctnnb1^flx(ex3)^, Ctnnb1^iGOF^, Ctnnb1^lox(ex3)^, Ctnnb^llox(ex3)^, Ctnnb^lox(ex3)^, GOF	KO, cKO	Whole body; intestines	(Harada et al. 1999)
ΔN90β-catenin-GFP		Introduction of ΔN90β-catenin-GFP	lowered levels of stabilized ∆90β-catenin in neural precursors	(Chenn and Walsh 2002)
*Ctnnb1*^*tm1Mmt*^ + Brn4-Cre		KO, cKO	Whole body; tissue from neuronal cell crest	(Zechner et al. 2003)
*Ctnnb1* ^*Bfc/+*^	Gena123	Thr653Lys substitution in the 12th armadillo repeat at the C-terminal end of the protein	Whole body	(Tucci et al. 2014)
APC^flox^+ CamKIIα-Cre		cKO	Whole body; excitatory neurons	(Pirone et al. 2017)
*Ctnnb1*^*ex3*/ex3^ + Foxd1-Cre		cKO	hypothalamic neuronal subtypes	(Newman et al. 2018b)
*Ctnnb1*^*tm1Mmt*^ + CamKIIα-Cre		cKO	excitatory neurons	(Alexander et al. 2020)
*Ctnnb*^*1em1V*^ + Pdgfb-iCre	Ctnnb1^floxedexon3^	cKO	retina	(Zhu et al. 2021)

KO-knockout; cKO-conditional knockout

Chenn and Walsh created a mouse model that overexpresses an N-terminally truncated form of the β-catenin protein, which is fused to green fluorescent protein (GFP) at the C-terminus (ΔN90β-catenin-GFP) in neuroepithelial precursors (Chenn and Walsh 2002). This form of β-catenin is constitutively stabilized, independent of Wnt signaling, as the GSK3 phosphorylation sites that normally target the protein for degradation in the absence of Wnt are absent. ΔN90β-catenin-GFP is capable of interacting with endogenous β-catenin and E-cadherin and can activate transcription by binding the TCF/LEF cofactors. The authors discovered that transgenic embryos at E15.5. have massively enlarged brains, wherein the brain cortex area is severely increased, although the thickness of the cortex is not altered. The same observance was noted at E17.5. Researchers found that β-catenin regulates cerebral cortical size, as its activity influenced the expansion of neuroepithelial precursors lining enlarged lateral ventricles (Chenn and Walsh 2002). This aligns with findings from Zechner et al. (2003), where β-catenin overexpression increased the size of the developing nervous system (Zechner et al. 2003).

The deletion of exon 3 in the *Ctnnb1* gene produces a β-catenin protein missing the N-terminal sequence, which is crucial for its degradation (Harada et al. 1999). Exon three encodes serines and threonine which are phosphorylated by glycogen synthase kinase 3b (GSK3b) (Guo et al. 2007) leading to subsequent degradation of beta catenin. This leads to the activation of the Wnt signaling pathway through a gain-of-function mutation in the *Ctnnb1* gene. Similar increase in nuclear localization of β-catenin protein can be also observed in Shank3 deficient mice (Qin et al. 2018), that also exhibit autism spectrum like disorders.

Harada et al. demonstrated that mating *Ctnnb1*^*Δex3*^ heterozygotes to mice, expressing Cre recombinase (Ck19-Cre) in the intestines resulted in offspring developing adenomatous intestinal polyps, confirming that the exon3 mutation can drive cancer development (Harada et al. 1999).

By matting β-cat^loxEx3^ mice with Brain4 transcription factor belonging to POU3 family promotor Brn4-Cre expressing mice (Ahn et al. 2001), Zechner et al. showed that mice expressing a stabilized β-catenin protein have an enlarged nervous system. Increased proliferation of progenitor cells in the spinal cord, forebrain, midbrain, and hindbrain led to an enlarged spinal cord, expanded ventricular zone in the embryos, and an increase in midbrain mass (Zechner et al. 2003). The same Brn4-Cre mice were mated with β-cat^floxEx3 – 6/floxEx3 – 6^ to generate a conditional LoF mouse model that exhibited diminished sizes of the nervous system, thus using these mouse models help researchers confirming the importance of the β-catenin protein in neuronal development.

Tucci et al. reported identification of several new *de novo* mutations in individuals with severe intellectual disability (Tucci et al. 2014). By employing chemical mutagenesis (Russell et al. 1979; Stottmann and Beier 2014) a whole body gain-off function mouse model was generated with a unique phenotype resulting from a gain in Wnt-related function but also a dominant-negative effect on adhesion through reduced affinity for membrane-associated cadherins. The mouse was named batface, due to distinctive craniofacial dysmorphology (*Bfc*; MGI:2656734) and exhibited specific morphological, behavioral, molecular and physiological traits that resemble the symptoms of CTNNB1 syndrome. The mutation was again lethal in homozygosity. The Batface mutation results in Thr653Lys substitution in the highly conserved 12th armadillo repeat at the C-terminal end of the protein. Prominent facial features in Bfc include a shortened nasal length, an increased interorbital distance, and an extended bregma-to-bony ridge length. Due to the position of the mutation, hippocampal cadherin-catenin interactions are reduced by 46%. MRI analysis of the mice revealed several brain abnormalities, including a shortened anteroposterior axis, enlarged dorsoventral and medial-lateral axes, and increased brain size and gray matter. Gyrification of the cerebral cortex was absent, and the thalamus, striatum, and globus pallidus were slightly larger, with some animals showing corpus callosum hypoplasia. Hippocampal neurons exhibited reduced dendritic branching compared to wild-types. Behavioral and physical assays revealed deficits in sensorimotor gating, motor function, and vocalization. The mutant mice showed impaired prepulse inhibition, while their acoustic startle response remained unchanged. Motor and cognitive impairments were evident in rotarod (shorter time to fall), water maze, and fear conditioning tests, indicating learning, memory, and motor deficits.

The adenomatous polyposis gene (APC) functions as a tumor suppressor and a key negative regulator of β-catenin turnover in the Wnt signaling pathway (Mohn et al. 2014). Conditional deletion of the APC gene in mice (APC cKO, using CamKIIα-Cre mice) thus also represents a gain-of-function mouse model in terms of beta catenin activity and shows characteristic symptoms of dysfunction of the β-catenin pathway. Neonatal APC cKO mice exhibit flexion-extension movements, motor spasms, and abnormal electroencephalographic activity, while adult animals display spontaneous electroclinical seizures (Pirone et al. 2017), all of which are indicative of autism spectrum disorder (ASD).

Another gain-of function mouse model, developed by crossing *Ctnnb1*^*ex3*/ex3^ mice with *Foxd1*-Cre expressing animals (Newman et al. 2018b) exhibits severe hyperplasia of the prethalamus and hypothalamus, along with expanded expression of specific posterior and premammillary hypothalamic markers. Foxd1 (a forkhead domain transcription factor) plays a crucial role in the terminal differentiation of hypothalamic neuronal subtypes (Newman et al. 2018a). Additionally, this model showed significant disruption in the development of the pituitary gland, determined by MRI.

Alexander et al. developed a gain-of function mouse model (Alexander et al. 2020) where they upregulated β-catenin in the presence of APC allocated to excitatory neurons. They conditionally overexpressed a stabilized truncated β-catenin by deleting the degradation domain. CamKIIα-Cre mice (Rios et al. 2001) were mated with β-cat^loxEx3^ mice (Harada et al. 1999). Excessive β-catenin expression led to both behavioral and molecular alterations, including reduced social interest and increased repetitive behaviors. Classic three-chamber social test revealed reduced social interactions. In performed open field test no motor changes were observed for the mutant animals. In the marble burying test the mice with overexpressed β-catenin buried less marbles, also the time of the marble interaction was increased, suggesting a repetitive stereotypical behavior. Researchers observed decreased parvalbumin levels and changes in the expression of several genes identified as potential autism risk factors in humans, confirming that this mouse model could be suitable for studying ASD related to CTNNB1 syndrome, but lacking motor deficits as seen in CTNNB1 syndrome patients.

Another clinical symptom in CTNNB1 syndrome patients is also exudative vitreoretinopathy, a retinal disease, characterized by the abnormal retinal angiogenesis, leading to incomplete peripheral retinal vascularization and ischemia that may eventually result in retinal detachments (Huang et al. 2023). Using CRISPR Cas nickase system (Ran et al. 2013) *Ctnnb1*^*fledExon3/+*^ mouse were generated by using the same design as Harada et al. (Harada et al. 1999); two loxP sites were placed up- and downstream of the exon3 of the *Ctnnb1* gene. By crossing these mice with Pdgfb-iCre–transgenic mice (Claxton et al. 2008), targeted expression in the vascular endothelium was achieved, resulting in increased β-catenin protein accumulation in retinal endothelial cells. This led to delayed superior retinal blood vessel growth and defects in vertical vascular growth into deeper retinal layers, a characteristic feature of FEVR (Zhu et al. 2021). This mouse model provides a valuable tool for studying visual impairments associated with CTNNB1 syndrome but lacking other CTNNB1 syndrome characteristics.

**Fig. 2:**
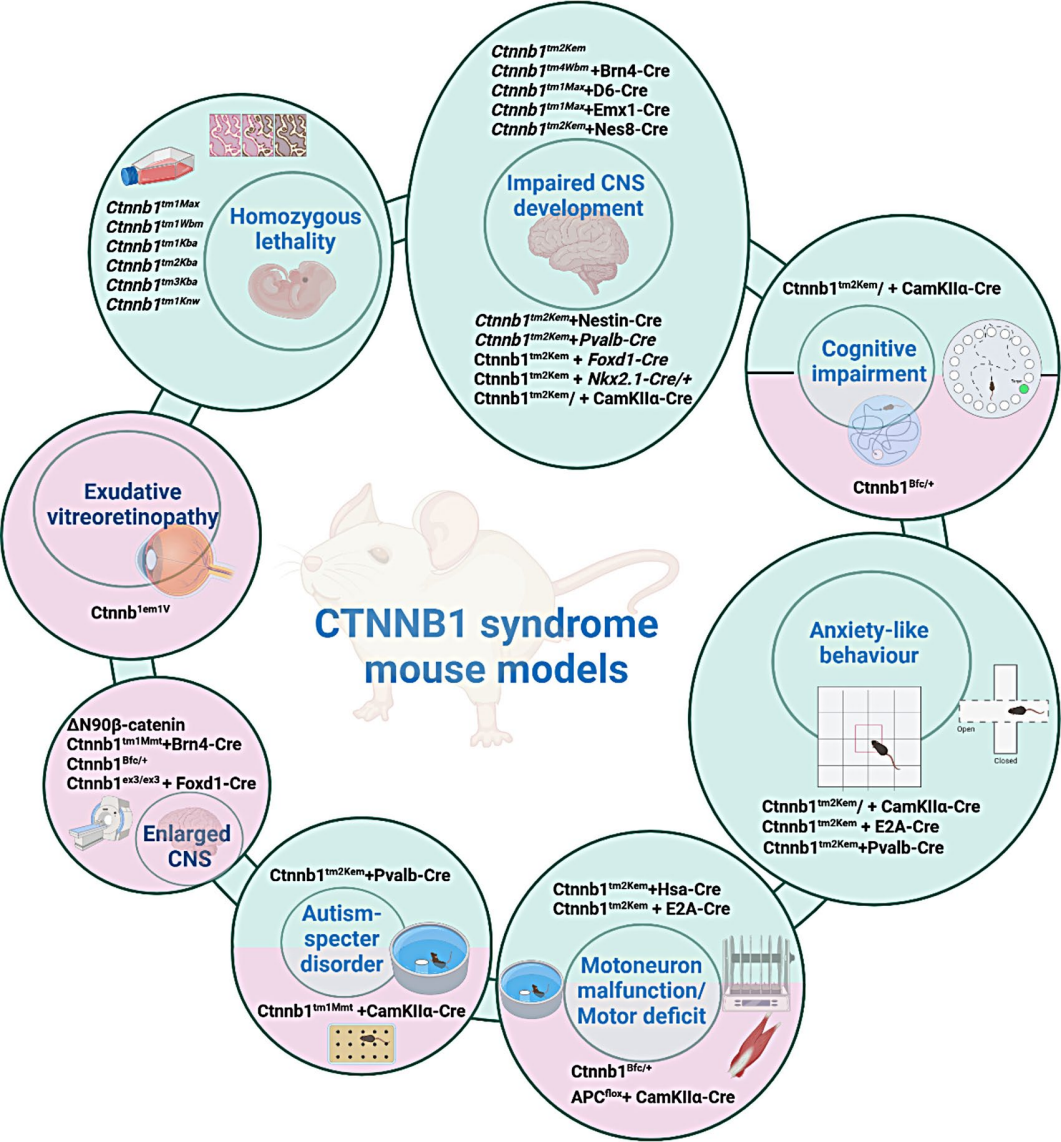
Overview of mouse models portraying specific traits correlated to the CTNNB1 syndrome. The mouse models (listed in black) are grouped with respect to traits that mimic symptoms of the CTNNB1 syndrome (listed in blue within the embeded circles). Green color indicates LoF mouse models, whereas pink indicates GoF models

## Conclusion

Several *Ctnnb1* mouse models have been described to date, wherein the majority of research uses whole body or conditional C*tnnb1* deletion (Haegel et al. 1995; Huelsken et al. 2000; Brault et al. 2001), generated by the Cre-loxP technology. Following the initial discoveries of gain-of-function mutations in *Ctnnb1* linked to oncogenesis (Harada et al. 1999; Chalamalasetty et al. 2016; Arnold et al. 2020; Loesch et al. 2022; AmeliMojarad et al. 2023; Cai et al. 2024), numerous *de novo* mutations have been identified and linked to CTNNB1 syndrome, a severe neurodevelopmental disorder. While gain-of-function mouse models primarily reflect hyperactive β-catenin signaling associated with oncogenesis and certain behavioral phenotypes, loss-of-function models highlight the critical role of β-catenin in neurodevelopment and associations with significant cognitive and motor impairments (Tucci et al. 2014; Kuechler et al. 2015; Alexander et al. 2024). By utilizing various tissue-specific Cre recombinase expression systems (Zechner et al. 2003; Pirone et al. 2017; Newman et al. 2018b), the effects of β-catenin alterations have been studied in a spatio-temporal manor, determining the important role of β-catenin in organ and body development, yet these mouse model lack specific CTNNB1 syndrome patient characteristics and are not suitable to use them as a mouse model to validate potential therapeutic solutions. Also, certain mouse models (using e.g. muscle, retina specific Cre etc.) exhibit only one characteristic of CTNNB1 syndrome patients and can be used to study the pathogenesis of that tissue-specific change but they lack general (motor, cognitive) impairments, seen in CTTNB1 syndrome patients and therefore these mouse models are not appropriate in the process of new drug development. β-catenin is crucial for cell junctions and signaling, significantly influencing motor function and neurodevelopment. This has been confirmed by numerous studies employing specific conditional knockouts to demonstrate the vital role of the β-catenin/Wnt pathway in neuronal organogenesis (Zechner et al. 2003; Newman et al. 2018b; Wickham et al. 2019). The function of *Ctnnb1* gene has been highlighted not only in the brain but also in other tissue and organs with presented effects that are not tightly connected to CTNNB1 syndrome per se but provide valuable data on β-catenin function (Grigoryan et al. 2008). In the context of LoF models, this review focuses particularly on models affecting brain and muscle tissues, as these are most relevant to studying CTNNB1 syndrome but as stated before they lack the plethora of CTNNB1 syndrome and cannot be used as a model to determine the potential therapy efficiency. Additionally, mice exhibiting a phenotype with visual impairments (Zhu et al. 2021) could be valuable for CTNNB1 syndrome studies as visual function problems are present in some patients.

Certain *Ctnnb1* mouse models mimic key features of the syndrome, including intellectual disability, motor deficits, and autism-like behaviors, making them essential tools for studying disease pathogenesis and drug discovery (Fig. 2), yet when using them it has to be considered that that they are not adequate for all CTNNB1 syndrome symptoms. Based on the published research it is notable that haploinsufficent mouse model, as shown by Alexander et al. (Alexander et al. 2024) exhibit the needed phenotype alterations compared to wild-type animals to use them in the process of new drug development. Homozygous animals are not viable, demonstrated by several researchers, using conditional and full body knock outs, therefore heterozygous animals (matted to appropriate Cre expressing mouse line) should be used in the process of new therapeutics discovery and researchers have to bear that in mind when developing suitable mouse models. Notable models, such as those derived by Tucci et al. and Alexander et al. demonstrate ASD phenotypes, anxiety-like behavior, and motor deficits, all hallmark symptoms of CTNNB1 syndrome (Tucci et al. 2014; Alexander et al. 2024), therefore are very useful in determining the efficiency of possible therapeutic approaches, but certain precautions should be considered as one mouse model cannot cover all traits of CTNNB1 syndrome, therefore researcher have to use them in a sound manner and rely on the data obtained from the genotype-phenotype study and history study of generating new mouse models. These models are of significant value for evaluating novel therapies, such as gene therapy, RNA-based treatments (e.g., antisense oligonucleotides), and small molecule repurposing (Alexander et al. 2024). Although no approved treatment currently exists for CTNNB1 syndrome, several potential therapeutic approaches have been proposed, such as using lithium (Stambolic et al. 1996) or GSK-3α/β inhibitors (Mao et al. 2009; Alexander et al. 2024) to modulate β-catenin/Wnt signaling. The two most advanced therapeutic approaches currently being explored are AAV9-mediated gene replacement therapy, spearheaded by the CTNNB1 Foundation (still in progress of the development) and GSK-3α/β inhibitors employed by Alexander et al. which have shown promising preclinical data in reversing the disease phenotype in mouse models, yet further research is still needed to fully conclude the therapeutic value of these approaches. The careful development and thorough phenotyping of appropriate mouse strains are of utmost importance for advancing drug discovery, highlighting the critical importance of selecting the right mouse models to study specific diseases and identify potential treatments.

## Data Availability

No datasets were generated or analysed during the current study.
